# Effect of menstrual cycle phase on physiological responses in healthy women at rest and during submaximal exercise at high altitude

**DOI:** 10.1038/s41598-024-79702-7

**Published:** 2024-11-13

**Authors:** Guia Tagliapietra, Tom Citherlet, Antoine Raberin, Nicolas Bourdillon, Bastien Krumm, Benjamin J. Narang, Guido Giardini, Vincent Pialoux, Tadej Debevec, Grégoire P. Millet

**Affiliations:** 1 Institute of Sport Sciences, Faculty of Biology and Medicine, University of Lausanne, Lausanne, 1015 Switzerland; 2 Faculty of Sport, University of Ljubljana, Ljubljana, 1000 Slovenia; 3 Department of Automatics, Biocybernetics and Robotics, Jožef Stefan Institute, Ljubljana, 1000 Slovenia; 4 Mountain Medicine and Neurology Center Valle D’Aosta, Regional Hospital, Aosta, 11100 Italy; 5 Interuniversity Laboratory of Motor Biology, Rockefeller Faculty of Medicine, University of Lyon, Lyon, 69008 France

**Keywords:** Acute high-altitude exposure, Cardiorespiratory, Net efficiency, Female, Ovarian hormone fluctuations, Post-exercise recovery, Physiology, Cardiology, Endocrinology, Medical research

## Abstract

As more women engage in high-altitude activities, understanding how ovarian hormone fluctuations affect their cardiorespiratory system is essential for optimizing acclimatization to these environments. This study investigates the effects of menstrual cycle (MC) phases on physiological responses at rest, during and after submaximal exercise, at high-altitude (barometric pressure 509 ± 6 mmHg; partial pressure of inspired oxygen 96 ± 1 mmHg; ambient temperature 21 ± 2 °C and relative humidity 27 ± 4%) in 16 eumenorrheic women. Gas exchange, hemodynamic responses, heart rate variability and heart rate recovery (HRR) were monitored at low altitude, and then at 3375 m on the Mont Blanc (following nocturnal exposure) during both the early-follicular (EF) and mid-luteal (ML) phases. Significant differences were observed between low and high-altitude in ventilation, heart rate and cardiac output. Resting ventilation (15.2 ± 1.9 vs. 13.2 ± 2.5 L.min^-1^; *p* = 0.039) and tidal volume (812 ± 217 vs. 713 ± 190 mL; *p* = 0.027) were higher during EF than ML at high-altitude. These differences between EF and ML were no longer evident during exercise, with comparable responses in oxygen uptake kinetics, cycling efficiency and HRR. The MC had negligible effects on physiological responses to high-altitude. An individualized approach, tailored to each woman’s specific responses to hypoxia across the MC, may be more beneficial in optimizing high-altitude sojourns than general guidelines.

## Introduction

An increasing number of women are exposed to high altitude for sport, work, mountain tourism, leisure and air travel. Notably, women represent 33% of American Alpine Club outdoor climbers, 40% of Everest trekkers and over 40% of Swiss Alpine Club members^1^. However, rapid ascent to 2500 m or above raises the risk of developing high-altitude illnesses (HAIs), including acute mountain sickness (AMS), cerebral and/or pulmonary edema^2^. The cardiorespiratory system plays a crucial role in acclimatization to high altitude^3,4^, which is essential for minimizing or even preventing these illnesses. Therefore, there is a growing emphasis on understanding the effect of the menstrual cycle on physiological responses to acute hypobaric hypoxia in females for preserving their health and optimizing their acclimatization, sojourns, physical activity, post-exercise recovery and safety at high altitude.

Despite their significant participation, women remain underrepresented in medical and applied sports physiology research, comprising only 39% of total participants in studies published in three leading sports science journals, with fewer than 13% of these studies focusing exclusively on women^5^. Results of studies conducted on men are not necessarily equally applicable to women given the anatomical, physiological and endocrinological differences between sexes^6^. In particular, eumenorrheic women experience cyclic hormonal fluctuations across the menstrual cycle. Estrogen levels rise during the follicular phase, reaching their peak just prior to the luteinizing hormone surge that triggers ovulation followed by the luteal phase, during which both estrogen and progesterone first increase, and then decrease in the absence of fertilization^7^.

At sea level, ovarian hormones have been shown to influence the cardiorespiratory system^8^. Progesterone is known to have a stimulatory effect on respiratory function, increasing the rate and depth of breathing, while estrogen may enhance the effects of progesterone on ventilation ($\dot V$_E_)^9^. Indeed, resting $\dot V$_E_ and core body temperature are higher during the luteal compared to the follicular phase^10^. However, it remains unclear whether the menstrual cycle affects resting $\dot V$_E_ at high altitude^6^, an important question to explore given the potential pathophysiological implications. Importantly, the increase in $\dot V$_E_ during the luteal phase, may result in an elevation in carbon dioxide output ($\dot V$CO_2_) and a subsequent reduction in the partial pressure of end-tidal carbon dioxide (P_ET_CO_2_) (hypocapnia) and a respiratory alkalosis. Moreover, hypocapnia may lead to cerebral hypoperfusion and impair cognitive function^11^. Therefore, understanding the effect of the menstrual cycle on ventilatory responses, particularly during acute exposure to hypobaric hypoxia, is crucial for clarifying how hormonal fluctuations influence health under hypoxic conditions. Similarly, research findings diverge regarding the impact of ovarian hormones on $\dot V$_E_ during maximal or submaximal exercise both in normoxic^12,13^ and hypoxic^14,15^ conditions. Interestingly, the latter of the two hypoxic studies, which focused on high-altitude native women, observed modest changes in $\dot V$_E_ between the follicular and the luteal phases during submaximal exercise, and no significant effects of the menstrual cycle on oxygen uptake ($\dot V$O_2_)^15^. However, these results should not be generalized to low altitude-residing women undergoing acclimatization. Contrasting results are also found in the literature regarding $\dot V$O_2_ during maximal and submaximal exercise. Specifically, one study conducted at night in normoxia reported higher $\dot V$O_2_ values and decreased cycling efficiency in the luteal phase during a 15-min exercise at 70% of their maximal $\dot V$O_2_, attributing these changes to an increased core body temperature and metabolic rate, driven by elevated progesterone levels^16^. Conversely, another investigation has reported no significant changes in maximal $\dot V$O_2_ across the menstrual cycle, with $\dot V$O_2_ remaining consistent between phases at a given constant workload, thereby leading to an unchanged cycling efficiency^12^. Furthermore, $\dot V$O_2_ responses during both peak and submaximal exercise to exhaustion were not affected by cycle phase, either at sea level or during hypobaric hypoxia exposure^14^. Discrepancies between studies may arise from differences in design, including variations in exercise intensity and duration, timing (e.g., day vs. night), environmental conditions (e.g., normoxia vs. hypobaric or normobaric hypoxia) and methods employed to assess $\dot V$O_2_. Additionally, participants’ fitness levels, hormonal profiles and the criteria used for menstrual phase determination can contribute to variation in findings. $\dot V$O_2_ kinetics were not affected by the menstrual cycle phase at sea level^17^.

Regarding cardiovascular responses, previous studies observed higher blood pressure (BP) at the onset of menstruation^18^ and increased heart rate (HR) during the luteal phase^19^. In contrast, cardiac output (CO) and stroke volume (SV), when normalized to body surface area, were similar between the early follicular (EF) and mid-luteal (ML) phases^20^. Furthermore, available data associated the decrease in heart rate variability (HRV) during the luteal phase with elevated progesterone levels^21^. Hormonal fluctuations along with changes in autonomic regulation during the menstrual cycle, such as higher sympathetic activity in the luteal phase^22^, may affect post-exercise heart rate recovery (HRR). However, examinations of parasympathetic tone markers of HRV and HRR indices revealed no discernible differences between the cycle phases^22^. Furthermore, a recent study on endurance-trained athletes found no interaction between menstrual cycle phase and recovery time for HR^23^ after high-intensity interval exercise. Whether these menstrual cycle-induced modulations are preserved at high altitude is less clear, particularly in healthy untrained females. An investigation conducted in acute hypobaric hypoxia observed consistent catecholamine levels across the menstrual cycle, but higher BP and HR during the luteal phase^24^. No differences between cycle phases were found in the hypoxic cardiac response during exercise^25^. Nevertheless, $\dot V$O_2_ kinetics, HRV, cycling efficiency and HRR, have not yet been explored at terrestrial high altitude during two different phases of the menstrual cycle. This is particularly important given that differential physiological responses (e.g., $\dot V$_E_, tidal volume (V_T_) and HR) have previously been observed between normobaric hypoxia and hypobaric hypoxia^26^.

To the best of our knowledge, no studies have investigated the impact of ovarian hormones on physiological responses to terrestrial high altitude at rest and during exercise during two distinct phases of the menstrual cycle using a within-subject study design. Given that altitude exposure imposes additional strain on the cardiorespiratory system, it is imperative to determine whether menstrual cycle phases alter responses to such conditions. This knowledge could inform tailored strategies for females exposed to a reduction in the partial pressure of inspired oxygen (PiO_2_). Therefore, the present investigation aimed to scrutinize whether any physiological differences exist at high altitude between the EF and the ML phases, both at rest and during submaximal exercise in healthy women. Emerging evidence reveals that physiological differences between menstrual phases, if present, are small^27^. Although the impact of hypoxia may overwhelm any subtle changes observed in normoxia^28^, a higher hypoxic ventilatory response has been reported during the luteal phase compared to the follicular phase^10^. In line with these findings, we hypothesized that significant differences in ventilatory responses would be observed between the two phases at rest. Conversely, we hypothesized that other physiological responses (e.g., cycling efficiency, $\dot V$O2 kinetics and HRR) would not exhibit significant alterations during exercise or post-exercise recovery, as there is insufficient evidence to suggest that these parameters are influenced by hormonal fluctuations.

## Methods

### Participants

Sixteen healthy and relatively untrained eumenorrheic women provided written informed consent to their voluntary participation in this study. They were selected according to the following inclusion criteria: aged 18 to 43 y, with regular menstruation (cycle length between 21 and 35 days, with no reported irregularities such as amenorrhea, anovulation or oligomenorrhea), physically active and with a body mass index ≤ 27 kg·m^− 2^. Over a 6-month period, menstrual cycle-related inclusion criteria for all participants were monitored through individual interviews conducted via telephone or email. Hormone concentrations were confirmed via post-hoc analysis of blood samples. Participants who used medications that could influence their hormonal responses, including hormonal contraception within three months prior to participation, were excluded. Participants were also asked to abstain from alcohol consumption, intensive physical activity within 24 h prior to the tests and prolonged altitude/hypoxia exposure within three months prior to the study, i.e., more than one night at ≥ 2000 m. None of the participants were taking any medication (e.g., acetazolamide) and all were free from any cardiorespiratory, neurological and hematological diseases. The experimental protocol was approved by the Ethical Committee of the Local Health Unit of the Aosta Valley Region (06/05/2021.0038781.I). The study was conducted in compliance with the latest guidelines outlined in the Declaration of Helsinki.

### Experimental protocol

Each participant took part in three experimental trials. They first underwent baseline assessments at low altitude (1224 m; Courmayeur, Italy; barometric pressure (Pb) 663 ± 1 mmHg; PiO_2_ 128 ± 0 mmHg), followed by a high-altitude (3375 m; at the Torino hut on the Mont Blanc chain, Courmayeur, Aosta Valley, Italy, Pb 509 ± 6 mmHg; PiO_2_ 96 ± 1 mmHg) crossover study involving a one-night stay and physiological tests during the EF (3 ± 1 days after menses onset) and the ML (6 ± 2 days before menses onset). These two phases demonstrate unique hormonal profiles. While EF is characterized by low levels of estrogen and progesterone, ML features a peak in these hormones, allowing for a comprehensive assessment of how menstrual cycle affects adaptation to high-altitude. On average, the duration between the two phases at high altitude was 15 ± 9 days. Of the 16 women, six reached high altitude first during EF and then during ML. At the visit conducted at low altitude, the 16 participants were stratified into two subgroups based on their ovarian hormone concentrations: nine were attributed to the follicular phase subgroup (F) and seven to the luteal phase subgroup (L). Low-altitude measurements were performed before the two high-altitude exposures, ensuring that participants were familiarized with the testing protocol and pre-emptively addressing any potential carryover effects of altitude exposure on the control assessments. Participants reached the Torino hut by cable car in 15–20 min for their two high-altitude trials. BP and HR were measured at rest on the left arm in a sitting position both at low altitude as well as 6 and 15 h after arrival at high altitude using a digital sphygmomanometer (M2, Omron Healthcare, Hoofddorp, The Netherlands). Based on preliminary results from our laboratory and on previous articles^25,29^, power output was set at 1.2 W·kg^− 1^, using the body mass recorded during the low-altitude trial to ensure absolute workload-matched exercise bouts across all visits. This intensity induces a steady-state necessary for accurate measurement of cycling efficiency, while minimizing the risk of fatigue, excessive stress and exacerbation of AMS^30^.

### Ventilatory and pulmonary gas exchange parameters

Respiratory frequency (Rf), V_T_, $\dot V$_E_, $\dot V$O_2_, $\dot V$CO_2_ and P_ET_CO_2_ were continuously monitored, breath by breath, using a metabolic cart (Quark, Cosmed, Rome, Italy) at rest and during exercise. Measurements were conducted at low altitude as well as 17 h after arrival at high altitude. The ventilatory equivalents for O_2_ and CO_2_ were expressed as $\dot V$_E_/$\dot V$O_2_ and $\dot V$_E_/$\dot V$CO_2_, respectively. Rest was defined as sitting on the bike without movement. Prior to each experiment, the device was calibrated using a 3-L syringe at various flow rates in accordance with the manufacturer’s guidelines. Subsequently, O_2_ and CO_2_ analyzers were calibrated using gas mixtures of known concentrations. Mean values of respiratory variables were calculated as the average over the final 60 s of both the resting period and the exercise bout. $\dot V$_E_ data included 16 subjects, while all variables within which O_2_ was required encompassed only 13 subjects due to a faulty sensor.

### Ventilatory work and respiratory muscle O2 consumption

The work of breathing (Wb) and respiratory muscle O_2_ consumption ($\dot V$RMO_2_) were assessed as previously described^3,1^ using the following equations for hyperventilation [1] and exercise [2], respectively:

1 $$Wb{\text{ }}\left( {kg\cdot{m^{ - 1}}\cdot{{\min }^{ - 1}}} \right) = 0.287 - 0.0212{\text{ }}{V_E} + 0.00287{\text{ }}{V_E}^2$$

2 $$Wb{\text{ }}\left( {kg \cdot {m^{ - 1}} \cdot {{\min }^{ - 1}}} \right) = - 0.251 + 0.0382{\text{ }}{V_E} + 0.00176{\text{ }}{V_E}^2$$

3 $$\dot VRM{O_2}\left( {mL \cdot {{\min }^{ - 1}}} \right) = 34.9 + 7.45{\text{ }}Wb$$

### Cycling efficiency

During the 6-min steady-state submaximal cycling exercise, net efficiency was calculated as previously described^32^ using the following equation:

$$W/\left( {E - {E_{rest}}} \right) \times 100$$

where W is the mechanical power and E-E_rest_ represents the metabolic power above rest.

Mean values were derived from the last 60 s of exercise.

### Oxygen uptake kinetics during the on- and off-phases of submaximal exercise

The $\dot V$O_2_ data were resampled at 1 Hz and then fitted with a mono-exponential function using a computerized non-linear regression technique to model the kinetics of both the on- and off-transients of $\dot V$O_2_, as previously described^33^. Analyses were conducted in MATLAB (v. R2019a, MathWorks, Natick, MA, USA). The parameters of interest were the amplitude (A), calculated as the difference in $\dot V$O_2_ values between the baseline and the steady-state, and the time constant (τ) representing the time from the onset of exercise to the point where the $\dot V$O_2_ curve reaches 63% of its final amplitude during the on-phase, and the τ of the exponential decay after exercise cessation during the off-phase.

### Cardiac hemodynamics

HR, SV and CO were continuously monitored at rest and during the submaximal exercise at low altitude and 17 h after arrival at 3375 m, using transthoracic impedance (Physioflow Enduro, Manatec Biomedical, Paris, France). BP was measured using the same method during exercise. Data were exported at 1 Hz, and 60-s arithmetic means were calculated at the end of the rest and exercise phases of the experimental protocol.

### Heart rate variability

HRV was measured at low altitude and 15 h after reaching high altitude, upon awakening, with an empty bladder, and following an 11-hour fasting period. The inter-beat interval (R-R interval) was recorded for 5 min using a chest belt (Polar H10, Kempele, Finland) connected to the Polar Sensor Logger mobile application (v. 0.25, Jukka Happonen, Helsinki, Finland), which operates via the Polar SDK (v. 3.3.2). HRV analyses were conducted using MATLAB (v. R2019a, MathWorks, Natick, MA, USA) employing a fast Fourier transform and the Welch power spectrum density estimate in the low-frequency (LF, 0.04–0.15 Hz; in ms^2^) and high-frequency bands (HF, 0.15–0.40 Hz; in ms^2^), and the root mean square of the successive differences (RMSSD; in ms), following the guidelines of the Task Force of the European Society of Cardiology and the North American Society of Pacing and Electrophysiology^34^. In addition to this, HRV analyses included the normalized HF (nHF, calculated as the HF/(LF + HF) ratio) and LF (nLF, LF/(LF + HF)) powers.

### Heart rate recovery

Post-exercise HRR was evaluated at low altitude and ~ 18 h after arrival at high altitude, with the participant in a passive seated position on a chair positioned adjacent to the cycle ergometer. The assessment commenced immediately after the conclusion of the 6-min submaximal exercise, and the transition from exercise to sitting was completed in less than 4 s. The analyses of HRR were conducted on MATLAB (v. R2019a, MathWorks, Natick, MA, USA) and involved multiple procedures. These included the delta between the final HR recorded during exercise and HR after 1 min (∆HR1min), 2 min (∆HR2min) and 3 min (∆HR3min) of recovery, acquisition of the HR decay τ by fitting the 10-min post-exercise HRR to a first-order exponential decay curve (HRRτ), and the semilogarithmic regression analysis of the first 30 s of HRR (T30) as previously proposed^35^. The RMSSD was computed for 30-s successive windows (RMSSD30s) through the 10-min recovery phase, serving as an index of post-exercise parasympathetic reactivation, and area under the curve (AUC, in s^2^) was determined by the logarithmic trapezoidal method providing an overall magnitude of RMSSD across time. HRV analyses conducted during the final 5 min of the recovery period – considered as a steady signal – included: RMSSD_5–10 min_, the power density in the HF band and the LF band (HF_5 − 10 min_, and LF_5–10 min_, respectively) as well as HFnu_5–10 min_, and LFnu_5–10 min_.

### Venous blood sampling and analysis

At low and high altitude, 6 mL of venous blood was sampled from the antecubital vein. Samples were centrifuged for 10 min at 3500 rpm, and the obtained plasma and serum were aliquoted into microtubes and frozen at -80 °C until analysis. Serum concentrations of estradiol and progesterone were measured to verify menstrual phase. Hormone levels were quantified using competitive enzyme-linked immunosorbent kits (Estradiol (E2) ELISA kit, MyBioSource, San Diego, CA, USA and progesterone (P4) ELISA kit, Abnova, Taipei City, Taiwan, respectively). The intra-assay coefficient of variation (CV) was 5.9% for estradiol and 7.2% for progesterone. The inter-assay CV was 7.8% for estradiol and 6.7% for progesterone. The same method was performed ~ 16 h after arrival at high altitude following a 12-hour fasting period.

### Statistical analysis

An a priori power analysis using G*Power software (v.3.1, G*Power software, Düsseldorf, Germany) was conducted to determine the appropriate sample size. Based on data from previous studies^13,14,19^ on the effects of menstrual cycle phase on cardiorespiratory responses and arterial O_2_ saturation, between 4 and 16 subjects were required to yield the targeted analysis power of 1-β = 0.8 with α = 0.05.

After assessing normality of data distributions with the Shapiro-Wilk test, differences between F and L groups under low altitude conditions were evaluated using a Student’s t-test for unpaired data. In addition to this, Levene’s test for equality of variances was employed to determine whether the assumption of equal variances was respected. When this assumption was violated, Welch’s t-test was used. Since no differences were observed between F and L subgroups at low altitude, except when indicated otherwise in the results, low-altitude data were pooled. After assessing normality of data distributions using the Shapiro-Wilk test, differences in means between low-altitude, EF and ML measurements were evaluated using a within-subjects ANOVA. When the normality assumption was violated, the Friedman non-parametric test and the Conover’s post hoc tests were used instead. In addition to this, Mauchly’s test was employed to verify the assumption of sphericity. When this condition was not met, the Greenhouse-Geisser correction was applied if the Greenhouse-Geisser epsilon (ε) values were < 0.75. Otherwise, the Huynh-Feldt correction was applied. The Holm post-hoc test was used to explore specific differences between conditions when a significant effect was detected.

Statistical significance was established at *p* < 0.05. Quantitative variables are presented as mean ± SD. All measurements and analyses were conducted on the 16 participants, except mentioned otherwise in methods or tables. All statistical analyses were performed using JASP (v. 0.18.3).

## Results

Participants’ baseline characteristics are displayed in Table 1.

**Table 1:** Baseline participants’ selected physical characteristics.

Participants’ characteristics	
Age (years)	33 ± 7
Height (cm)	166 ± 7
Body mass (kg)	60 ± 10
Body mass index (kg·m^− 2^)	22.7 ± 3.2
Cycle length (days)	27 ± 2

Values are Mean ± SD. N = 16.

No differences were observed between EF and ML in Pb (507 ± 6 vs. 510 ± 5 mmHg) and P_i_O_2_ (95.8 ± 1.3 vs. 96.4 ± 1.0 mmHg).

Order effects were assessed by comparing visit 1 to visit 2 at high altitude, using the same statistical approaches as for the main outcomes, and no significant differences were observed.

### Ovarian hormones

Mean estradiol and progesterone levels, presented in Table 2, are within previously reported normal ranges for eumenorrheic women^36^. Progesterone demonstrated significantly higher levels in L compared to F (*p* = 0.002) and during ML compared to EF (*p* < 0.001).

**Table 2:** Serum concentrations of progesterone and estradiol.

	Low altitude		High altitude	
	Follicular phase subgroup	Luteal phase subgroup	Early follicular phase	Mid-luteal phase
Estradiol (ng·L^− 1^)	45.3 ± 22.2	38.1 ± 31.4	46.9 ± 24.5	56.7 ± 23.1
Progesterone (ng·mL^− 1^)	3.72 ± 1.49	**7.89 ± 2.50** ^**a**^	3.81 ± 2.01	**7.86 ± 2.59** ^**ab**^

Values are Mean ± SD. ^***a***^ *p* < 0.05 for differences with F; ^***b***^*p* < 0.05 for differences with EF. N = 16.

### Measurements at rest

Tables 3 and 4 display key cardiorespiratory parameters evaluated at rest at both low and high altitude depending on the menstrual cycle phase of interest. Respiratory parameters and peripheral oxygen saturation (SpO_2_) exhibited significant differences between low and high altitude (Table 3**)** with the exception of $\dot V$O_2_, which remained consistent across the three conditions.

**Table 3:** Ventilatory, gas exchange parameters and peripheral oxygen saturation at rest at low altitude and 17 h post arrival at high altitude.

	Low altitude	High altitude	
		Early follicular	Mid-luteal
Rf (breaths·min^− 1^)	18.0 ± 2.7	**20.3 ± 4.6** ^**#**^	**20.3 ± 3.9** ^**#**^
V_T_ (mL)	646 ± 120	**812 ± 217** ^**##**^	**713 ± 190** ^*^
$\dot V$_E_ (L·min^−1^)	10.8 ± 1.8	**15.2 ± 1.9** ^**###**^	**13.2 ± 2.5** ^## **^
$\dot V$O_2_ (mL·min^−1^·Kg^−1^)	5.57 ± 1.20	6.20 ± 0.99	5.47 ± 1.14
$\dot V$CO_2_ (mL·min^−1^)	278 ± 53	**341 ± 80** ^**#**^	292 ± 53
$\dot V$_E_/$\dot V$O_2_	28.2 ± 3.2	**37.3 ± 6.9** ^**##**^	**35.2 ± 5.8** ^**##**^
$\dot V$_E_/$\dot V$CO_2_	34.8 ± 4.1	**42.3 ± 7.2** ^**#**^	**40.6 ± 5.3** ^**#**^
P_ET_CO_2_ (mmHg)	30.6 ± 2.7	26.9 ± 5.8	**26.1 ± 3.0** ^**#**^
SpO_2_ (%)	98.9 ± 0.9	**92.4 ± 2.3** ^**###**^	**91.2 ± 3.1** ^**###**^
Wb (kg·m^− 1^·min^− 1^)	0.401 ± 0.068	**0.638 ± 0.119** ^**###**^	**0.517 ± 0.148** ^# *^
$\dot V$RMO_2_ (mL·min^−1^)	37.9 ± 0.5	**39.7 ± 0.9** ^**###**^	**38.8 ± 1.1** ^# *^

Values are Mean ± SD. ^#^*p* < 0.05, ^##^*p* < 0.01, and ^###^*p* < 0.001 for differences with low altitude. *p < 0.05, **p < 0.01, ***p < 0.001 for differences with early follicular. Rf, respiratory frequency; V_T_, tidal volume; $\dot V$_*E*_, minute ventilation; $\dot V$O_*2*_, oxygen uptake; $\dot V$CO_*2*_, carbon dioxide production; $\dot V$_*E*_/$\dot V$O_*2*_, ventilatory equivalent for O_*2*_; $\dot V$_*E*_/$\dot V$CO_*2*_, ventilatory equivalent for CO_*2*_; *P*_*ET*_*CO*_*2*_, end tidal partial pressure of carbon dioxide; SpO_*2*_, peripheral oxygen saturation; Wb, work of breathing; $\dot V$RMO_*2*_, respiratory muscle O_*2*_ consumption. N = 16, except for gas exchange parameters at high-altitude where *n* = 13.

**Fig. 1:**
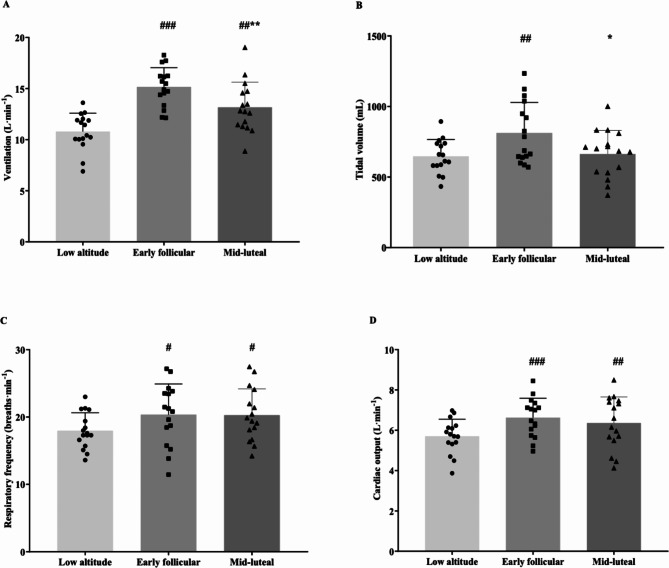
Main ventilatory and cardiac responses at rest at low and high altitude. Values are Mean ± SD.^*#*^*p* < 0.05, ^*##*^*p* < 0.01, and ^*###*^*p* < 0.001 for differences with low altitude. *p < 0.05, **p < 0.01.

Similarly, HR, SV and CO were significantly different between low and high altitude. Furthermore, $\dot V$_E_ (*p* = 0.039) and V_T_ (*p* = 0.026) were significantly higher in EF compared to ML (Fig. 1). However, the other cardiorespiratory variables investigated, including HRV, presented no significant differences between EF and ML (Table 4).

**Table 4:** Hemodynamics and heart rate variability at rest at low altitude and 17 h post arrival at high-altitude.

	Low altitude	High altitude	
		Early follicular	Mid-luteal
HR (bpm)	63.7 ± 10.6	**75.3 ± 14.2** ^**#**^	**78.5 ± 13.0** ^**##**^
SBP (mmHg)	96.7 ± 13.7	103.6 ± 11.2	99.0 ± 7.3
DBP (mmHg)	67.0 ± 6.9	73.6 ± 6.9	72.8 ± 6.5
SV (mL)	80.4 ± 13.2	**68.6 ± 8.1** ^**###**^	**66.6 ± 11.5** ^**###**^
CO (L·min^− 1^)	5.7 ± 0.9	**6.6 ± 1.0** ^**###**^	**6.4 ± 1.3** ^**##**^
RMSSD (ms)	42.3 ± 25.8	42.7 ± 27.1	36.0 ± 18.7
LF (ms^2^)	961 ± 694	1182 ± 1605	914 ± 1029
HF (ms^2^)	916 ± 568	776 ± 647	620 ± 592
nLF (n.u.)	55.4 ± 18.0	55.8 ± 18.9	54.7 ± 22.6
nHF (n.u.)	44.6 ± 18.0	44.3 ± 18.9	45.3 ± 22.6

Values are Mean ± SD. ^*#*^ *p* < 0.05, ^*##*^*p* < 0.01, and ^*###*^*p* < 0.001 for differences with low altitude. HR, heart rate; SBP, systolic blood pressure; DBP, diastolic blood pressure; SV, stroke volume; CO, cardiac output; RMSSD, root-mean-square difference of successive normal R–R intervals; LF, power in low frequency range; HF, power in high frequency range; nLF, normalized LF power; nHF, normalized HF power. *N* = 16.

### Physiological responses during exercise

Analyses between F and L subgroups conducted at low altitude revealed significant differences in ∆HR1min (46.5 ± 7.8 vs. 37.6 ± 5.4 bpm; *p* = 0.025), ∆HR2min (56.4 ± 5.8 vs. 44.6 ± 7.6 bpm; *p* = 0.005) and ∆HR3min (58.8 ± 5.4 vs. 47.3 ± 9.9 bpm; *p* = 0.014). Similarly, HF_5 − 10 min_ (246 ± 129 vs. 94 ± 126 ms^2^; *p* = 0.041), LFnu_5 − 10 min_ (0.63 ± 0.17 vs. 0.85 ± 0.07; *p* = 0.012) and HFnu_5 − 10 min_ (0.37 ± 0.17 vs. 0.15 ± 0.07; *p* = 0.012) were significantly different between F and L phases, respectively.

At high altitude, the cardiopulmonary responses during submaximal intensity exercise, performed 17 h after arrival were unaffected by cycle phase (Table 5), with the exception of the amplitude of $\dot V$O_2_ values during the on-transient response which exhibited an 8% difference between EF and ML (*p* = 0.043). Similarly, cycling efficiency remained consistent between EF and ML.

**Table 5:** Ventilatory, gas exchange, peripheral oxygen saturation and hemodynamics during submaximal exercise at low and high altitude.

	Low altitude	High altitude	
		Early follicular	Mid-luteal
*Cardiorespiratory parameters*			
Rf (breaths·min^− 1^)	30.3 ± 4.8	32.6 ± 5.5	32.3 ± 5.5
V_T_ (mL)	1490 ± 260	**1670 ± 310** ^**###**^	**1680 ± 350** ^**###**^
$\dot V$_E_ (L·min^−1^)	42.7 ± 8.5	**53.9 ± 13.2** ^**###**^	**53.5 ± 13.4** ^**###**^
$\dot V$O_2_ (mL·min^−1^·Kg^−1^)	23.6 ± 2.8	20.5 ± 5.5	21.9 ± 3.6
$\dot V$CO_2_ (mL·min^−1^)	1467 ± 201	1384 ± 381	1388 ± 216
$\dot V$_E_/$\dot V$O_2_	28.7 ± 4.7	**39.0 ± 9.4** ^**###**^	**37.2 ± 6.4** ^**###**^
$\dot V$_E_/$\dot V$CO_2_	31.0 ± 4.5	**38.5 ± 8.8** ^**###**^	**37.2 ± 4.8** ^**##**^
P_ET_CO_2_ (mmHg)	36.7 ± 4.7	**30.4 ± 6.0** ^**##**^	**30.7 ± 4.1** ^**#**^
HR (bpm)	138 ± 20	**148 ± 13** ^**###**^	**146 ± 1 5** ^**###**^
SBP (mmHg)	109 ± 12	105 ± 9	106 ± 10
DBP (mmHg)	71.9 ± 7.1	73.5 ± 6.7	72.1 ± 8.3
SV (mL)	99.4 ± 15.7	**83.7 ± 8.0** ^**###**^	**80.5 ± 11.5** ^**###**^
CO (L·min^− 1^)	13.7 ± 2.8	12.5 ± 1.7	**12.0 ± 2.4** ^**#**^
SpO_2_ (%)	94.1 ± 5.7	**87.2 ± 5.7** ^**#**^	89.0 ± 4.0
Wb (kg·m^− 1^·min^− 1^)	4.40 ± 2.07	**7.13 ± 3.20** ^**##**^	**7.22 ± 3.19** ^**###**^
$\dot V$RMO_2_ (mL·min^−1^)	67.2 ± 15.1	**87.4 ± 23.6** ^**###**^	**86.7 ± 23.7** ^**##**^
Cycling efficiency (%)	20.1 ± 1.9	21.7 ± 3.7	19.8 ± 1.2
RPE (6–20)	10.7 ± 2.6	11.9 ± 2.0	11.8 ± 1.5
$\dot V$O_*2*_*kinetics*			
A_on_ (mL·min^− 1^)	1018 ± 141	**916 ± 256** ^**##**^	996 ± 115 *
τ_on_ (s)	32.2 ± 6.2	**36.4 ± 7.7** ^**#**^	35.3 ± 7.4

Values are Mean ± SD.^*#*^ *p* < 0.05, ^*##*^*p* < 0.01, and ^*###*^ *p* < 0.001 for differences with low altitude. *p < 0.05 for differences with early follicular. Rf, respiratory frequency; V_*T*_, tidal volume; $\dot V$_*E*_, minute ventilation; $\dot V$O_*2*_, oxygen uptake; $\dot V$CO_*2*_, carbon dioxide production; $\dot V$_*E*_/$\dot V$O_*2*_, ventilatory equivalent for O_*2*_; $\dot V$_*E*_/$\dot V$CO_*2*_, ventilatory equivalent for CO_*2*_; *P*_*ET*_*CO*_*2*_, end tidal partial pressure of carbon dioxide; HR, heart rate; SBP, systolic blood pressure; DBP, diastolic blood pressure; SV, stroke volume; CO, cardiac output; SpO_*2*_, peripheral oxygen saturation; RPE, rate of perceived exertion,* A*_*on*_, amplitude of oxygen uptake values during the on-transient response; τ_*on*_, time constant of the on-transient pulmonary oxygen uptake kinetics. *N* = 16, except for gas exchange parameters at high-altitude where *n* = 13.

Moreover, the time constant of the on-transient pulmonary $\dot V$O_2_ kinetics (τ_on_) and the other parameters characterizing $\dot V$O_2_ kinetics during the off-phase of moderate-intensity exercise (Tables 5 and 6) exhibited no significant differences between EF and ML. No differences were observed in HRR parameters between EF and ML (Table 6).

**Table 6:** Postexercise oxygen uptake kinetics and heart rate recovery parameters in time and frequency domains at low altitude and 17 h post-arrival at high-altitude.

	Low altitude	High altitude	
		Early follicular	Mid-luteal
$\dot V$O_2_ kinetics			
A_off_ (mL·min^− 1^)	1089 ± 150	991 ± 248	1006 ± 134
τ_off_ (s)	40.1 ± 6.9	**45.1 ± 7.8** ^**#**^	**45.9 ± 6.8** ^**#**^
Heart rate recovery			
∆HR1min (bpm)	42.3 ± 8.0	40.5 ± 8.0	37.8 ± 5.1
∆HR2min (bpm)	50.9 ± 8.9	49.8 ± 6.1	46.0 ± 6.8
∆HR3min (bpm)	53.4 ± 9.6	54.1 ± 7.1	49.2 ± 8.9
HRRτ (s)	46.6 ± 24.0	48.1 ± 26.5	44.2 ± 23.9
A (bpm)	57.7 ± 11.3	56.4 ± 8.1	**51.2 ± 10.1** ^**#**^
T30 (s)	128 ± 43	157 ± 88	171 ± 79
AUC (s^2^)	13.9 ± 8.8	9.2 ± 6.5	8.1 ± 4.4
RMSSD_5–10 min_ (ms)	23.9 ± 14.8	16.2 ± 11.1	15.2 ± 8.6
LF_5–10 min_ (ms^2^)	417 ± 304	370 ± 465	333 ± 255
HF_5–10 min_ (ms^2^)	185 ± 145	150 ± 186	124 ± 163
nLF_5–10 min_ (n.u.)	71.7 ± 17.7	75.9 ± 14.3	77.1 ± 16.1
nHF_5–10 min_ (n.u.)	28.3 ± 17.7	24.1 ± 14.3	22.9 ± 16.1

Values are Mean ± SD. #*p* < 0.05 for differences with low altitude. Aoff, amplitude of oxygen uptake values during the off-transient response; τoff, time constant of the off-transient pulmonary oxygen uptake kinetics; ∆HR1min, absolute difference between the final heart rate at exercise completion and the heart rate recorded after 1 min of recovery; ∆HR2min, absolute difference between the final heart rate at exercise completion and the heart rate recorded after 2 min of recovery; ∆HR3min, absolute difference between the final heart rate at exercise completion and the heart rate recorded after 3 min of recovery; HRRτ, time constant of the heart rate decay; A, amplitude of the peak-to-baseline heart rate difference; T30, short-term heart rate time constant; AUC, the area under the curve; RMSSD_5–10 min_, root-mean-square difference of successive normal R–R intervals; LF_5–10 min_, power in low frequency range; HF_5–10 min_, power in high frequency range; nLF_5–10 min_,normalized LF power; nHF_5–10 min_, normalized HF power. N = 9.

## Discussion

The primary aim of the present study was to investigate whether potential physiological differences exist between EF and ML, both at rest and during submaximal exercise, at high altitude in healthy and relatively untrained women. To the best of our knowledge, this is the first study showing that cycling efficiency, $\dot V$O_2_ kinetics, HRV and HRR were not impacted by the menstrual cycle during acute high-altitude exposure, as initially hypothesized. Nevertheless, respiratory function appeared to be slightly affected by the cycle phase at rest.

Ovarian hormone levels were within previously reported normal ranges for eumenorrheic women^36^. Progesterone concentrations increased significantly during ML, with all participants exhibiting values above the 10 nmol·L^− 1^ (3 ng·mL^− 1^) threshold for the luteal phase and above 16 nmol·L^− 1^ (5 ng·mL^− 1^) for the mid-luteal phase^36^. Although estradiol levels were higher in ML compared to EF, the difference was not statistically significant, likely due to the high variability in hormone concentrations between subjects, which reduced the statistical power. Within the existing literature, definitive cut-off values for estradiol corresponding to distinct menstrual phases remain undefined due to substantial inter- and intra-individual variability in ovarian hormone concentrations^36^. Moreover, no consensus has been reached among researchers regarding the specific hormonal profiles associated with each phase of the menstrual cycle. In the present investigation, participants’ menstrual cycle lengths and related symptoms were monitored over a 6-month period preceding the commencement of the tests as well as during the study. Phases (i.e., EF and ML) were determined based on this individual data and hormone level verification through serum sample analyses.

As previously observed^2^, respiratory parameters (i.e., V_T,_ $\dot V$_E_) and SpO_2_ exhibited significant differences between low and high altitude at rest. In contrast, $\dot V$O_2_ remained consistent across conditions, corroborating earlier findings^28^.

Beyond the altitude-related alterations, the present study also observed differences in ventilatory responses across the menstrual cycle. Resting $\dot V$_E_ and V_T_ were significantly higher during EF compared to ML. Notably, Muza et al.^3,7^ reported a 0.11% difference in $\dot V$_E_ between the menstrual cycle phases, corresponding to a non-significant increase in the follicular compared to the luteal phase at 4300 m, using a between-groups design. These findings challenge the commonly reported progesterone-driven increase in respiratory depth (V_T_) at sea level^9^, suggesting that high-altitude may modify expected hormonal effects on respiratory function. Indeed, hypobaric hypoxia induces unique physiological adjustments, including changes in respiratory drive and pattern, oxygen availability, and acid-base balance^2^. Of interest is that ventilatory acclimatization was shown different between normobaric and hypobaric hypoxia^38,3,9^, reinforcing the relevance of the present study performed at terrestrial altitude. However, further research is necessary to elucidate the underlying mechanisms of these observations. Furthermore, the increase in $\dot V$_E_ without a corresponding rise in $\dot V$_E_/$\dot V$O_2_ suggests a potential elevation in metabolic demand or respiratory cost. This is further supported by the higher Wb and $\dot V$RMO_2_ during EF compared to ML. Although, $\dot V$O_2_ showed a non-significant increase between the two menstrual phases. The lower core body temperature in EF may reduce the metabolic demand and potentially compensate for the increased respiratory cost.

Contrary to resting conditions, no significant differences were found between EF and ML during submaximal exercise at high altitude in terms of $\dot V$_E_, $\dot V$O_2_, $\dot V$_E_/$\dot V$O_2_ and $\dot V$_E_/$\dot V$CO_2_. Thus, ventilatory efficiency, evaluated as $\dot V$_E_/$\dot V$CO_2_, remained unaffected by the menstrual-cycle phase. These results are consistent with previous findings where increased plasma progesterone in the ML phase of eight female lowlanders did not significantly affect ventilatory responses during submaximal exercise in a hypobaric chamber simulating 4300 m^14^. Exercise-induced respiratory drive may have overridden any hormone-mediated effects on $\dot V$_E_ measured at rest. Moreover, a recent study^40^ demonstrated that hypoxia induces a greater increase in $\dot V$_E_ during exercise compared to rest. This potentiation is mediated by neural mechanisms (e.g., medullary signal summation and sympathetic carotid chemoreceptor activation), rather than humoral factors (e.g., hypoxemia within the active skeletal muscle circulation or the release of metabolic by-products from skeletal muscles). Even if no differences in $\dot V$O_2_ kinetics between EF and ML have been observed in the current study, the time constant of the on- and off-transient pulmonary oxygen uptake kinetics were significantly slower at high altitude compared to low-altitude, as previously reported^41^.

Cardiac hemodynamics were unaffected by the menstrual phase at 1224 m and at 3375 m. Under normoxic conditions, previous investigations have reported a higher BP at the onset of menstruation than during the late follicular phase^18^. This is potentially due to elevated endogenous estrogen levels during the late follicular phase, which may exert vasodilatory effects by stimulating nitric oxide release or by acting directly on the vascular smooth muscle^42^. However, in the current investigation, BP remained stable across the menstrual cycle phases at both low and high altitude, in agreement with a previous study conducted in normoxia^43^. The stable BP observed during the two phases of the menstrual cycle at 3375 m could be attributed to the counterbalancing effects of progesterone opposing estrogen’s influence on vascular tone^44^ and to the cycle phases examined. In fact, our study did not investigate the late-follicular phase, which is characterized by the highest estrogen levels across the full cycle.

As previously observed^2^, SV significantly decreased, while HR significantly increased with altitude at rest and during the submaximal exercise, resulting in a higher CO. However, these variables were not influenced by the menstrual cycle phase at high altitude.

It was previously reported that HRV fluctuates across the menstrual cycle^21^. However contrasting results are also found in the literature, with either higher sympathetic activity^45^ or greater parasympathetic activity^46^ during the luteal phase. Regarding this, estrogen was shown to increase choline uptake and acetylcholine synthesis influencing parasympathetic tone^47^, whereas progesterone may increase sympathetic activity^48^. However, the effect of progesterone on the autonomic nervous system remains controversial. Interestingly, a recent study associated the HRV decrease during the luteal phase with the progesterone peak^21^. It is important to note that due to high inter-individual variations, within-subject designs are highly recommended. In the present study, HRV, assessed using a within-subject design, remained unchanged between EF and ML at high altitude, indicating that menstrual phase does not influence autonomic regulation in healthy and relatively untrained women during acute exposure to hypobaric hypoxia. Discrepancies between studies may result from interindividual variability in HRV, methodologies used for data processing, participants’ characteristics and the position adopted during the test. Additionally, methods used to identify cycle phases, hormone concentration variability, individual responsiveness to a given ovarian hormone level and hormone receptor expression can also contribute to variation in findings.

Previous research combining men and women indicates that as altitude increases, HRV tends to decrease, with a significant reduction observed at an elevation of 4600 m and above^49^. Similarly, in the current study investigating specific responses to high altitude in eumenorrheic women across the menstrual cycle, LF, HF, nLF and nHF were not significantly different between 1224 m and 3375 m.

Sympathetic hyperactivity or reduced cardiac vagal tone after exercise are associated with increased risk of cardiovascular disease^35^. It has been demonstrated that HRR from treadmill exercise until maximal exhaustion was unaffected by menstrual phase in normoxia^22^. However, HRR remains significantly under-investigated in eumenorrheic women. In the present study, differences in the ∆HR between the end of the exercise and specific recovery time points, i.e., 1 min, 2 min and 3 min after a submaximal exercise bout, have been observed between F and L subgroups at low altitude, suggesting a modulating effect of the menstrual cycle on the post-exercise parasympathetic reactivation. However, the HRRτ and T30 were not different between F and L. Moreover, a greater HF_5 − 10 min_was observed in the F subgroup, indicating increased parasympathetic activity from minutes 5 to 10 in the recovery period. It is important to acknowledge potential confounding factors, such as individual variability, particularly given that subjects in the two subgroups were different and that the sample sizes were small. At high altitude, HRR assessed using a within-subject design remained unchanged between EF and ML, suggesting that altitude may diminish the phase-dependent variations reported in normoxia. Overall, the menstrual cycle did not elicit any detectable change in BP, HR, HRV and HRR at high altitude, confirming our hypotheses.

The strengths of the present study are the monitoring of the menstrual cycle over a 6-month period prior to the beginning of the study including menstrual cycle symptoms, considering variations in cycle length within women and verifying the menstrual cycle phase using hormonal analyses. Additionally, we tested the same subjects at terrestrial high altitude during two different menstrual cycle phases and included 16 relatively untrained women not exposed to high altitude prior to the study, ensuring that tests were not confounded by previous hypoxic exposure and training status. Moreover, participants performed familiarization tests before acute exposure to high altitude. Finally, as recently advocated^26^, the values of Pb and P_i_O_2_ were recorded daily and there was no difference between EF and ML measurements, ensuring comparable environmental conditions.

Nevertheless, some limitations must be acknowledged relating primarily to logistical constraints. First, baseline assessments were conducted at low altitude (1224 m) rather than sea-level. Secondly, the majority of participants were inhabitants of Aosta Valley, permanently living at altitudes between 500 m and 1220 m. Some of them may have undergone an acclimatization to low altitude compared to lowlanders, potentially influencing the observed physiological responses during acute high-altitude exposure. However, the observed significant increases in $\dot V$_E_, HR and CO from low to high altitude suggest that participants were not pre-acclimatized. Furthermore, although the study aimed to maintain a randomized crossover design, logistical constraints resulted in less women reaching high altitude first during the EF than ML (6 vs. 10). However, order effect assessment provides some evidence that visit 1 and visit 2 did not differ, indicating that the sequence of visits is unlikely to have influenced the key outcomes of the study.

Overall, the present study provides novel insights into the complex relationship between menstrual cycle phases and physiological responses to acute high altitude in healthy and relatively untrained women. The present findings indicate that hormonal influences on cardiorespiratory function at 3375 m are small or insignificant. In fact, despite a higher $\dot V$_E_, underpinned by V_T_, during EF compared to ML at rest, $\dot V$O_2_ kinetics, cycling efficiency, HRV and HRR remained consistent across the menstrual cycle. This is in line with emerging evidence that reveals trivial or insignificant effects of the menstrual cycle on multiple physiological outcomes^14^. Consequently, there is currently very little evidence to aptly recommend a specific menstrual cycle phase for high-altitude travel. Healthy, non-athlete women likely do not need to adjust their acclimatization strategies based on their cycle phase. However, individual variability should still be considered, and rather than relying on general guidelines, it is advisable to adopt an individualized approach, considering each woman’s specific response to high-altitude across the menstrual cycle. Regardless of the menstrual phase, progressive acclimatization remains the primary advice to reduce the risk of severe AMS. In light of these findings, the increased representation of females in rigorous altitude/hypoxic research is strongly encouraged.

Further research is warranted to substantiate our findings and explore longitudinal responses to prolonged high-altitude exposure across multiple menstrual cycles in order to investigate the intra-subject variability that is known to be high^36^. Therefore, conducting a study at higher altitude and extending the duration of the exposure beyond 18 h would clarify the effect of the menstrual cycle on sub-acute acclimatization mechanisms.

## Data Availability

The data that support the findings of this study are available from the corresponding author upon reasonable request.
